# Penicilliosis in a Non-HIV Patient: A Case Report

**DOI:** 10.7759/cureus.37484

**Published:** 2023-04-12

**Authors:** Sai Chandra Hakeem, Kiran G Kulirankal, Ann Mary, Merlin Moni, Dipu T Sathyapalan

**Affiliations:** 1 Department of Internal Medicine, Amrita Institute of Medical Sciences and Research Centre, Kochi, IND

**Keywords:** penicilliosis, invasive fungal infections, itraconazole, liposomal amphotericin b, immunocompromised patient

## Abstract

A 68-year-old female, with a known case of mantle cell lymphoma, came with complaints of persistent cough with expectoration for three months, not responding to multiple courses of antibiotics. Bronchoscopy was done and bronchoalveolar lavage (BAL) culture revealed *Penicillium *species. She was started on IV liposomal amphotericin B for 14 days and then switched to oral itraconazole which showed a response to treatment. Early diagnosis of penicilliosis and prompt treatment are important as it is rare and associated with a high mortality rate.

## Introduction

*Penicillium *species are mainly opportunistic pathogens. They are generally observed in immunocompromised individuals, most often in patients with Human Immunodeficiency Virus (HIV) [1]. The only etiologic agent for this infection is a thermally regulated dimorphic fungus named *Talaromyces marneffei* (formerly *Penicillium*). It is most commonly reported in South East Asian countries like China, Thailand, Hong Kong, North Eastern India, and Taiwan. It is the most common opportunistic infection found in HIV-affected individuals after Tuberculosis and *Cryptococcus *[2].

Here, we report a case of a 68-year-old lady with a known case of mantle cell lymphoma. She came in with complaints of cough for the past three months. Despite being treated with multiple courses of antibiotics, her symptoms did not subside. Her sputum culture revealed *Penicillium *species. She was treated with IV liposomal amphotericin B for 14 days and later switched to oral itraconazole with which she had clinical resolution.

## Case presentation

A 68-year-old female with a known history of mantle cell lymphoma treated with six cycles of bendamustine and rituximab visited the outpatient department with complaints of progressive worsening of cough for three months.

Her cough was productive in nature and associated with white-colored mucoid sputum. There was no diurnal or positional variation in her cough. She had visited numerous hospitals and was treated with multiple courses of oral antibiotics over the last three months that provided her with no relief. She also complained of intermittent episodes of low-grade fever during this period and weight loss of 5 kgs over the past six months. In view of her immunocompromised state and chronicity of cough, her sputum was sent for AFB stain and GeneXpert to rule out tuberculosis, which came in as negative. Labs showed anemia (Hb-Hb-8.3, MCV-89), leucopenia (total counts - 3400, neutrophils - 74%), platelet - 2.2 lakhs, erythrocyte sedimentation rate (ESR) - 80. Her blood and urine cultures were sterile.

**Figure 1:**
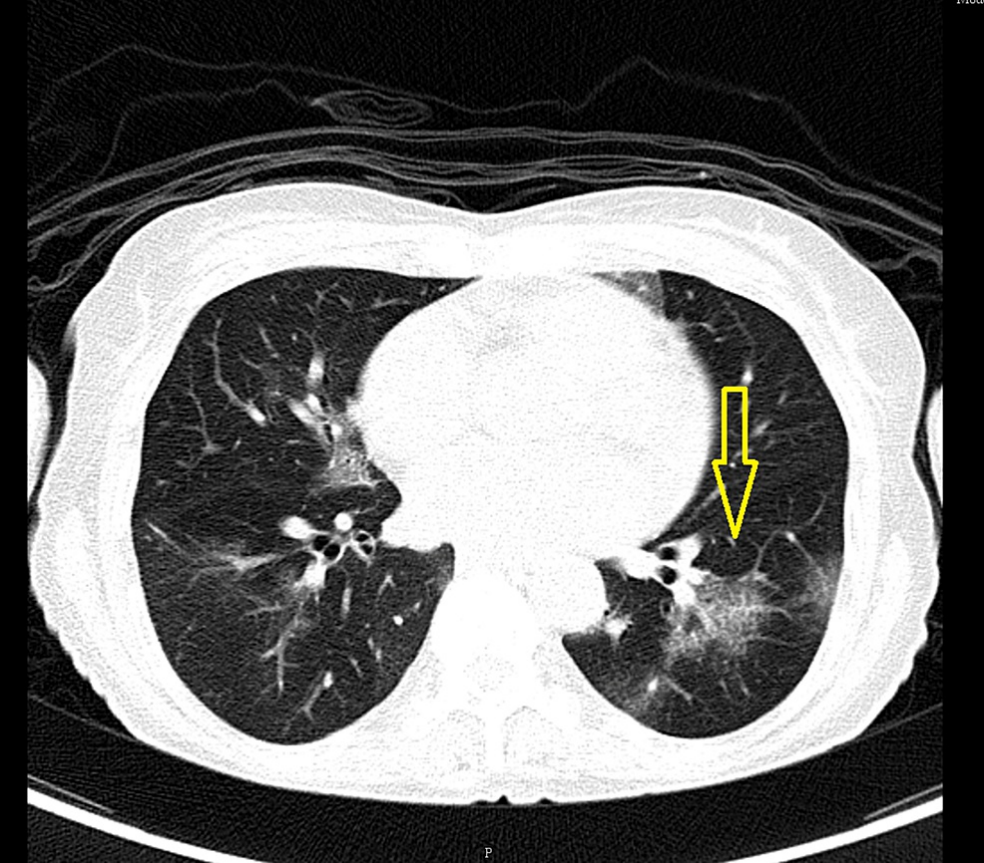
CT showing patchy ground glass opacity with interstitial thickening and subtle bronchiectasis changes in the left lower lobe and right middle lobe CT: computed tomography

While consulting hematology, a positron emission tomography-computed tomography (PET-CT) scan was performed and it revealed diffuse fluorodeoxyglucose (FDG) uptake in patchy ground glass opacities in bilateral lungs: probable active infection/inflammatory (Figure 1).

In view of the presence of ground glass opacities in bilateral lungs along with non-resolving cough in an immunocompromised individual, a bronchoscopy was performed and samples were sent for analysis. Bronchoalveolar lavage (BAL) samples were sent for *Mycobacterium tuberculosis* GeneXpert and AFB smear, which came back negative. Cytology revealed scattered neutrophils and a few lymphocytes. BAL culture grew *Penicillium *species.

She was diagnosed as having penicilliosis. Thereafter, she was started on injection of liposomal amphotericin B (5mg/kg) IV once a day for 14 days and then switched to oral Itraconazole 200mg twice a day for 10 weeks. She showed a good response to the treatment and her symptoms resolved.

## Discussion

Penicilliosis is a thermally dimorphic fungus. It is an endemic infection in South Asia. The first infection was discovered in 1973 in an individual with Hodgkin's lymphoma [3]. As the incidence of HIV increased, the number of penicilliosis cases also increased, thereby indicating it to be an opportunistic pathogen. It is a dimorphic fungus, existing in several forms such as mold at 25 degrees and yeast at 37 degrees centigrade. The reservoir includes wild bamboo rats. Humans are infected by inhalation of spores from the environment [4]. The disease can affect the respiratory tract, skin, intestinal tract, bones, and joints and may also disseminate across the entire body. Penicilliosis can affect the respiratory system. It is often confused with tuberculosis and patients are commonly treated with antitubercular drugs that do not respond and are considered as drug-resistant tuberculosis. Common clinical symptoms include cough, expectoration, dyspnea, and fever [5].

Kawila et al. in their study compared the clinical presentation between HIV-affected and non-HIV-affected patients and demonstrated that fever and splenomegaly are common in HIV patients, whereas bone and joint infection are common in non-HIV individuals [6].

Penicilliosis is becoming common nowadays even amongst non-HIV patients, especially in the case of patients with impaired cellular immunity like solid organs and hematopoietic organ transplant as well as in patients treated with anti-CD20 monoclonal antibodies [7].

The importance of this pathogen is that it is rarely considered in the differentials and is often confused with tuberculosis due to the close resemblance in symptoms. Therefore, often, people are also treated with antitubercular drugs and the symptoms do not subside in spite of prolonged treatment with antitubercular drugs [8].

The definitive diagnosis is by culture. However, a provisional diagnosis can be made from a smear. It grows on Sabaroud Dextrose Agar in four to seven days at 25 degrees. It is observed as a flat green surface on a dark red colony and microscopically shows a filamentous structure. Once this is done, yeast-to-mold conversion at 37 degrees should be demonstrated on brain heart infusion agar. Skin samples can be sent for polymerase chain reaction (PCR) testing. Galactomannan can show cross-positivity with this.

The diagnosis and prompt treatment are very important as it holds a mortality rate of 97% if the disease is untreated or diagnosed late [9]. The treatment consists of an induction phase and a consolidation phase. The duration of the induction phase is two weeks. Liposomal amphotericin B (3-5mg/kg/day) is the preferred drug of choice. The duration of the maintenance phase is 10 weeks. Oral itraconazole (200mg twice a day) is the drug of choice during the maintenance phase [10].

## Conclusions

Penicilliosis is an opportunistic infection observed in immunocompromised people. Its symptoms resemble that of tuberculosis and patients are diagnosed late due to a low index of suspicion, hence the mortality rate is high. Prompt diagnosis and early initiation of treatment are important to achieve favorable outcomes.
